# *Neospora caninum* Calcium-Dependent Protein Kinase 1 Is an Effective Drug Target for Neosporosis Therapy

**DOI:** 10.1371/journal.pone.0092929

**Published:** 2014-03-28

**Authors:** Kayode K. Ojo, Molly C. Reid, Latha Kallur Siddaramaiah, Joachim Müller, Pablo Winzer, Zhongsheng Zhang, Katelyn R. Keyloun, Rama Subba Rao Vidadala, Ethan A. Merritt, Wim G. J. Hol, Dustin J. Maly, Erkang Fan, Wesley C. Van Voorhis, Andrew Hemphill

**Affiliations:** 1 Center for Emerging and Re-emerging Infectious Diseases (CERID), Division of Allergy and Infectious Diseases, Department of Medicine, University of Washington, Seattle, Washington, United States of America; 2 Department of Biochemistry, University of Washington, Seattle, Washington, United States of America; 3 Institute of Parasitology, Vetsuisse Faculty, University of Berne, Berne, Switzerland; 4 Department of Chemistry, University of Washington, Seattle, Washington, United States of America; University at Buffalo, United States of America

## Abstract

Despite the enormous economic importance of *Neospora caninum* related veterinary diseases, the number of effective therapeutic agents is relatively small. Development of new therapeutic strategies to combat the economic impact of neosporosis remains an important scientific endeavor. This study demonstrates molecular, structural and phenotypic evidence that *N. caninum* calcium-dependent protein kinase 1 (*Nc*CDPK1) is a promising molecular target for neosporosis drug development. Recombinant *Nc*CDPK1 was expressed, purified and screened against a select group of bumped kinase inhibitors (BKIs) previously shown to have low IC_50s_ against *Toxoplasma gondii* CDPK1 and *T. gondii* tachyzoites. *Nc*CDPK1 was inhibited by low concentrations of BKIs. The three-dimensional structure of *Nc*CDPK1 in complex with BKIs was studied crystallographically. The BKI-*Nc*CDPK1 structures demonstrated the structural basis for potency and selectivity. Calcium-dependent conformational changes in solution as characterized by small-angle X-ray scattering are consistent with previous structures in low Calcium-state but different in the Calcium-bound active state than predicted by X-ray crystallography. BKIs effectively inhibited *N. caninum* tachyzoite proliferation *in vitro*. Electron microscopic analysis of *N. caninum* cells revealed ultra-structural changes in the presence of BKI compound 1294. BKI compound 1294 interfered with an early step in *Neospora* tachyzoite host cell invasion and egress. Prolonged incubation in the presence of 1294 interfered produced observable interference with viability and replication. Oral dosing of BKI compound 1294 at 50 mg/kg for 5 days in established murine neosporosis resulted in a 10-fold reduced cerebral parasite burden compared to untreated control. Further experiments are needed to determine the PK, optimal dosage, and duration for effective treatment in cattle and dogs, but these data demonstrate proof-of-concept for BKIs, and 1294 specifically, for therapy of bovine and canine neosporosis.

## Introduction

The apicomplexan parasite, *Neospora caninum*, is the leading cause of epidemic abortion in cattle and is also a frequent cause of neuromuscular diseases in dogs. A recent estimate of neosporosis-induced abortions in cattle from Argentina, Australia, Brazil, Spain, Canada, Mexico, New Zealand, United Kingdom and the USA suggests an annual economic impact in excess of 1.2 billion USD [1]. *N. caninum* related diseases have also been reported in other livestock species, including sheep, goats, horses and deer [2],[3] with canids such as dogs, wolves and coyotes being the definitive hosts [4],[5]. Therefore, neosporosis represents a major veterinary health and economic concern. Neoguard, a killed tachyzoite lysate and the only vaccine that has been marketed to date, was reported in one publication to reduce the abortion rate in cattle by approximately 50% [6] but does not induce protection against fetal infection in cattle. However, more recent trials revealed fewer efficacies in the field, and even suggested that vaccination may increase the risk of early embryonic death [7]. Thus, the vaccine has been taken off the market. The application of live attenuated vaccines, although potentially more efficacious, carries the risk of early embryonic death as a result of potential reversion to virulence [7]. Chemotherapy could provide a viable alternative if appropriate compounds are identified. Both *in vivo* and *in vitro* studies have been performed to determine efficacy of treatments with lasalocid, monensin, pirithrexim, pyrimethamine, clindamycin, robenidine and trimethoprim [8], artemisinin and artemisone [9], [10], depudecin [11], toltrazuril, and ponazuril [12] although none of these studies showed that treatments were actually effective in cattle. To date there is no approved vaccine and no approved treatment for cattle that are infected with *N. caninum*.

A study on prophylactic administration of toltrazuril in newborn calves suggests some degree of protection based on serological response data [13], but protection has never been proven. Treatment with prolonged administration of clindamycin or potentiated sulfa drugs (in combination with trimethoprim) were only successful in eliminating clinical signs in less than 40% cases of canine neosporosis [14]. Clearly, a new and more efficacious therapeutic is needed to reduce the economic impact of neosporosis on farmers and the global economy. We recently demonstrated that apicomplexan calcium-dependent protein kinases (CDPKs) are promising drug targets, as observed from excellent correlations between cell-activity and enzyme inhibition by compounds from a focused bumped kinase inhibitor (BKI) library as well as chemical-genetic validation [15]–[17]. *Toxoplasma gondii* CDPK1 (*Tg*CDPK1) (TGME49_101440) has been reported to be associated with host cell invasion processes [15], [18]. *Tg*CDPK1 has the smallest possible gatekeeper amino acid in the ATP binding site, glycine. Most BKIs have bulky C3 aryl substituents that project into a hydrophobic pocket adjacent to the glycine gatekeeper in the ATP binding site. BKIs selectively inhibit *Tg*CDPK1 but don't inhibit mammalian kinases because mammalian kinases have larger gatekeeper residues that block the entry of the bulky C3 aryl substitution into the adjacent hydrophobic pocket. *N. caninum* is similar to *T. gondii* in its ability to invade a large variety of cells [19]–[21] and their genomes are highly similar as well. The two parasites share a conserved core proteome, similar core biochemical processes, and 99% CDPK1 amino acid sequence identity including a glycine gatekeeper residue. Thus, it seems likely that BKIs, which efficiently inhibit host cell invasion in *T. gondii*, will also block *N. caninum* invasion and growth. However, it is necessary to test the function of *Nc*CDPK1 as divergence of functions or pathways may have occurred.

In this report, we describe the biochemical, structural, and biophysical aspects of *Nc*CDPK1 enzyme and the nanomolar potency of BKIs in the inhibition of *Nc*CDPK1. The effect of BKI inhibitors on *N. caninum* tachyzoite proliferation and development were determined. Seven of the tested BKIs exhibit low-to-mid nanomolar activity against *N. caninum* proliferation *in vitro*, making them good candidates for therapeutic investigation. A therapeutic oral trial with a BKI, 1294, demonstrated marked reduction of *N. caninum* cerebral parasite burden in mice, thus demonstrating proof-of-concept for neosporosis therapy. Thus, our studies define a strategy for the development of potent and safe *Nc*CDPK1 inhibitors that could form the basis of a novel neosporosis treatment regimen.

## Results

### In vitro activity of BKIs against NcCDPK1 enzyme activity and N. caninum proliferation

**Figure 1:**
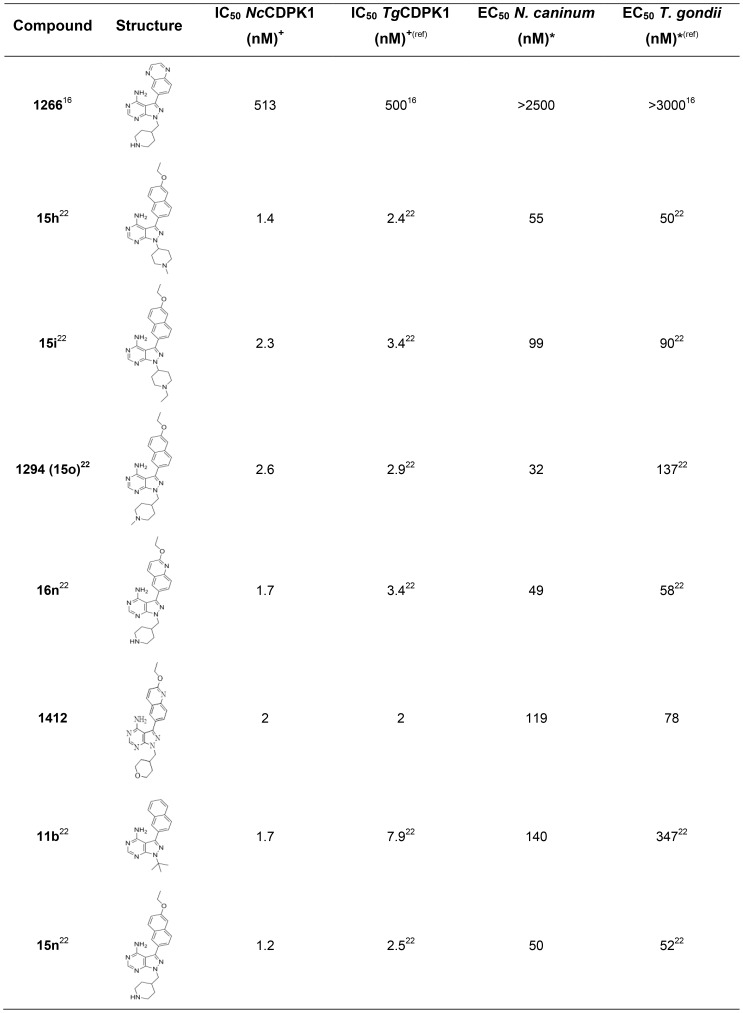
*In vitro* activity of BKIs against *Nc*CDPK1 activity and *N*. *caninum* proliferation. ^+^IC_50_ represents the concentration that inhibits protein kinase activity by 50%. *EC_50_ represents the concentration that inhibits cell growth by 50%.

The potency of 8 BKIs against recombinant *Nc*CDPK1 kinase activity was evaluated. Seven of the BKI compounds inhibited *Nc*CDPK1 enzyme with IC_50_ in the range of 1.2 to 2.6 nM, while one of them, BKI 1266 [16], had a much higher IC_50_ at 513 nM (Figure 1). The observed IC_50_ values are closely related to previously published *Tg*CDPK1 inhibition data for these same compounds [22]. The EC_50_ values of the inhibitors to block *N. caninum* growth were determined. The seven BKIs with 1 to 3 nM IC_50s_ for inhibiting *Nc*CDPK1 enzyme activity inhibit the growth of *N. caninum* tachyzoites *in vitro* with EC_50_ values in the range of 49 to 140 nM. Compound 1266 [16], with the higher IC_50_ of 513 nM, was unable to block *N. caninum* proliferation *in vitro*, even at the highest concentration tested (2500 nM). Though there are not many BKIs tested yet, there is a good correlation between potency *in vitro* towards *Nc*CDPK1 inhibition and *N. caninum* growth. None of these compounds showed mammalian cell cytotoxicity when assayed against human lymphoblastoid cell, CRL 8155, and none had off-target inhibition of small gatekeeper (threonine) human protein kinases [22]. Our data shows 1294 (Figure 1) had a 2.6 nM IC_50_ against *Nc*CDPK1 enzyme and 32 nM EC_50_ against *N. caninum* tachyzoites. Compound 1294 has previously been reported to exhibit profound *in vitro* and *in vivo* activity against *Cryptosporidium parvum* [17] cell growth and have excellent malaria transmission blocking properties [23]. Pharmacokinetic data with 1294 demonstrates favorable absorption with low clearance, supporting daily oral administration, and low toxicity in mammalian cells and rodents [23]. Efficacy against *N. caninum* tachyzoites in cell culture points to another anti-apicomplexan application for 1294 justifying it for broad spectrum drug development.

### Crystallographic confirmation of inhibitor binding modes

**Figure 2:**
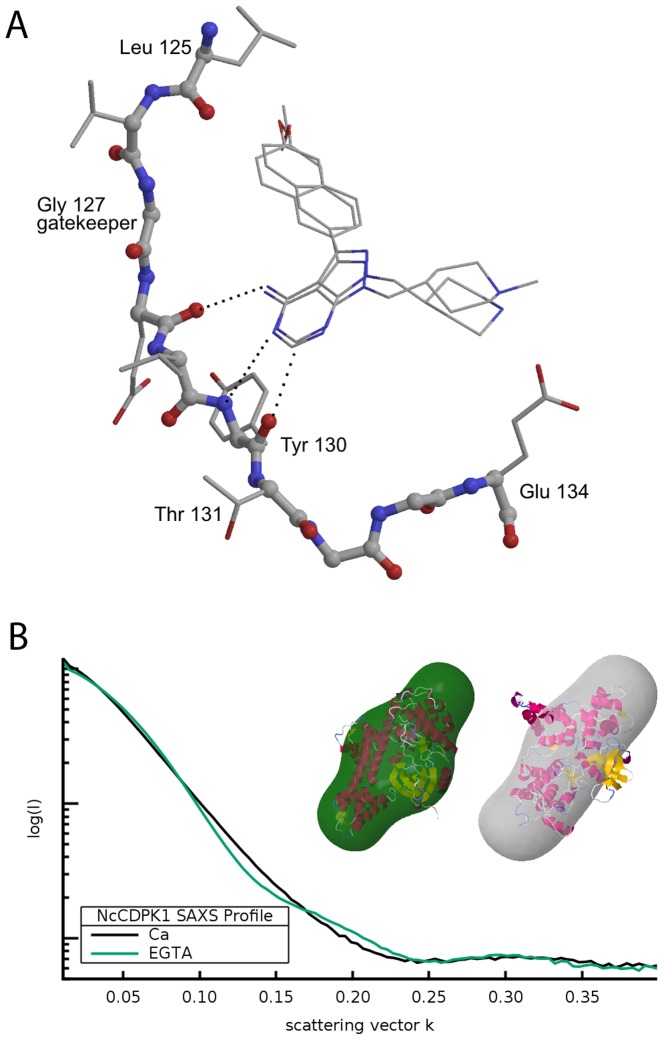
a: Binding pose of pyrazolopyrimidine analogues in complexes *Nc*CDPK1. The crystallographically observed binding poses of inhibitors RM-1-132 (15n) and 1294 (15o). The bulky naphthalene substituent is accommodated by the presence of a small (glycine) gatekeeper residue. The piperidine substituents extend into the ribose binding pocket of the kinase active site. The pose and interactions of the PP scaffold with the protein backbone are highly consistent for members of this series of BKI compounds ^24^. b: Conformational states of *Nc*CDPK1 in solution using SAXS. The SAXS profiles measured for the low calcium (green) and high calcium (black) states of *NcCDPK1* in solution. The inset shows the corresponding low resolution 3D models generated from the respective profiles, superimposed onto crystal structures of the low calcium state of *NcCDPK1* (this work; PDB 4m97) and a high calcium state of *TgCDPK1* (PDB 4hx4). The crystallographic and SAXS models agree better for the low calcium state (left) than for the high calcium state (right). The model for the high calcium state derived from the observed SAXS profile (gray solid) is more extended, i.e. has a larger radius of gyration, than the crystal structure.

*NcCDPK1* is 96% sequence identical to the well-studied *TgCDPK1*. The active site environment of *Nc*CDPK1 differs at only amino acid position 123 having tyrosine rather than phenylalanine at *Tg*CDPK1 residue 124. This change does not alter the binding surface in the active site, but may act to slightly rigidify the active site conformation by introducing a new hydrogen bonding interaction between residues Tyr 123 and Glu 88. The observed binding pose of the pyrazolopyrimidine (PP) scaffold and the R1 substituent “bump” in the inhibitor complexes studied here is entirely consistent with that seen previously for PP-scaffold inhibitors bound to the *T. gondii* homolog (Figure 2A, Figure S1 and S2 in File S1). Compound 1266 [16] is approximately 200 fold less effective at inhibiting CDPK1s relative to other BKIs shown in the study probably due to its distinctive quinoxaline R1 ring system rather than the common naphthalene, quinoline, or isoquinoline R1 group. A three-dimensional (3-D) model of CDPK1 with 1266 predicted that the quinoxaline nitrogen at position 9 (N9) is oriented toward the carbonyl oxygen of residue valine 78 (3.3 Å distance). This would have been quite favorable for additional hydrogen bonding if the N9 was hydrogen. However, a quinoxaline nitrogen may not have a proton at pH values above 1, making this an unfavorable interaction because the carbonyl oxygen of residue valine 78 and the N9 pay a price in solvation energy by facing each other rather than solvent waters. Data from this study confirmed that the model for the structural basis of potency and specificity developed for the optimization of potential anti-toxoplasmosis drugs based on this scaffold is also applicable to optimization of anti-neosporosis drugs [22], [24]. The pose of the piperidine R2 substituent in the current structures is similar but not identical to that seen in *Tg*CDPK1 complexes with other inhibitors containing piperidine ring substituents at this position, which in turn are not identical to each other. This argues for flexibility in the orientation of the piperidine substituents even after binding of these nanomolar inhibitors, and allows additional chemical modifications of this group if necessary to further optimize PK/AMDET properties.

### Ca^2+^-dependent conformational state in solution

The structure of *Nc*CDPK1 as seen in both the apo and inhibitor-bound crystals is consistent with the “inactive” forms previously seen in calcium-free crystal structures of CDPK1 homologs from *C. parvum* (ApiDB_CryptoDB:cgd3_920) and *T. gondii* (TGME49_101440). In this conformational state the C-terminal Ca^2+^-binding domain forms a compact structure that occludes the active site of the N-terminal kinase domain, thus preventing the recognition or phosphorylation of substrate proteins. Intracellular regulation of CDPK activity is believed to be mediated by a radical conformational rearrangement that occurs in the presence of sufficiently high concentrations of Ca^2+^. This is supported by previous crystal structures of calcium-bound states of *C. parvum* and *T. gondii* homologs [25], but direct measurement of CDPK conformational states in solution has not been previously reported. Therefore we undertook characterization of the low- and high- (Ca^2+^) conformational states of *Nc*CDPK1 in solution using small angle X-ray scattering (SAXS).

As predicted from crystal structures of the low and high calcium conformations of *TgCDPK1*, the solution X-ray scattering profiles for *Nc*CDPK1 in the two states are significantly different, as are the derived conformational models (Figure 2B). However while there is good agreement between the SAXS and crystallographic models for the low-(Ca^2+^) state, both the radius of gyration and the envelope of the SAXS reconstruction indicate a high-(Ca^2+^) state in solution that is less compact than was captured in previous crystal structures of homologous CDPKs with bound calcium.

The scattering profile for the low-(Ca^2+^) state in solution indicates a 29.1±2.3 Å radius of gyration (R_g_). Direct reconstruction of the implied conformational state is consistent with the low-(Ca^2+^) state seen in the current *Nc*CDPK1 crystal structures and with the previously reported calcium-free crystal structures of CDPK1 homologs. This agreement is also shown by the good correlation cc = 0.88 between the observed scattering curve and the curve predicted from the apo *Nc*CDPK1 crystal structure. The scattering profile for the high-(Ca^2+^) state in solution indicates a radius of gyration Rg = 35.1±1.8 Å, significantly larger than that observed for the low-(Ca^2+^) state. Direct reconstruction of the implied high-(Ca^2+^) conformational state in solution yields a model that is distinctly different from the reconstruction of the low-(Ca^2+^) state. The correlation between the observed scattering curve and that predicted from a crystal structure of the homologous calcium-bound *Tg*CDPK1 (PDB 3hx4) is cc = 0.83.

The most likely explanation for the less compact state observed for the high-(Ca^2+^) conformation of *Nc*CDPK1 as measured in solution when compared to that of *Tg*CDPK1 as seen in a crystal is that in solution the rearranged secondary structural elements of the Ca^2+^-binding domain exhibit sufficient flexibility for the protein to adopt a range of conformational states. Only a single relatively compact state from this range is favored, or stabilized, during formation of the crystal lattice. An equivalent discrepancy between the observed crystalline and solution states of the calcium-binding motifs in calmodulin is well-known [26]. It is also possible, although less likely, that there is a true difference between the calcium-bound states of *Tg*CDPK1, for which there is crystallographic data, and *Nc*CDPK1, for which there is only SAXS data. There are three sequence differences between *Nc*CDPK1 and *Tg*CDPK1 in the calcium-binding domain that might hypothetically influence the dynamics of the calcium-bound state. These two possible explanations are not mutually exclusive.

### Compound 1294 prevents *N. caninum* tachyzoite host cell invasion

**Figure 3:**
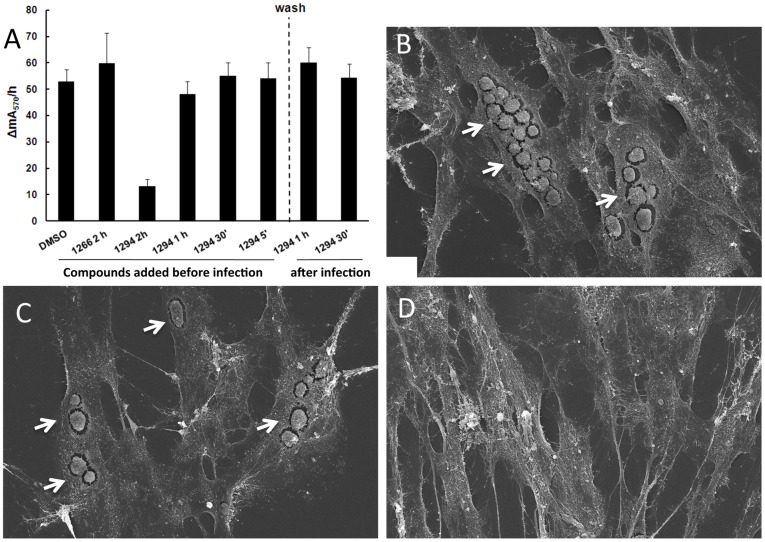
Compound 1294 interferes in host cell invasion. (A) Measurement of β-galactosidase activities in HFF during the invasion process. HFF monolayers were infected by incubating them with 2×10^4^ *N. caninum (b-Gal)* tachyzoites for 2 hours. Prior to this 2 hour invasion phase (timepoint 0′), tachyzoites were suspended either in DMSO (DMSO 0′), in 5 μM compound 1266 (1266 0′) or in 5 μM 1294 (1294 0′). In additional assays, compound 1294 was added at 60 min after initiation of invasion (1294 60′), 90 min (1294 90′) or 115 min (1294 115′) prior to removal of the drug by washing. Cultures were then incubated for an additional 60 min before β-galactosidase activity was measured. There was a significant decrease in β-galactosidase activity (t-test; p<0.001) when compound 1294 was added at the beginning of infection (1294 0′), but not when the compound was added at later timepoints, and no changes occurred when 1294 was added after the infection phase (1294 60′ post-infection. (B–D) Scanning electron micrographs of HFF infected with *N. caninum* for 3 days. Cultures were maintained in the absence of 1294 (B), in the presence of 2.5 μM 1294 added 2 h post-invasion (C), and in the presence of 2.5 μM 1294 added already at the time point of invasion (D). Cultures were processed for SEM analysis after 3 days. Note the presence of parasitophorous vacuoles in B and C (arrows), and respective absence in D.

In *T. gondii*, it has been postulated that the activity of BKIs is based on the inhibition of serine threonine kinase CDPK1 phosphorylation activity needed in part to regulate intracellular calcium induced activation of the glideosome system and apical secretion of micronemal transmembrane adhesins which engage the motor complex for successful invasion of the host cells [15], [27]. Analysis of *N. caninum* growth and proliferation when treated with compound 1294 over an infection time course of 2 hours suggests that it severely impairs the host cell invasion process (Figure 3A). Its greatest impact is associated with the early stage of the host cell-parasite interaction. When 1294 was added to cultures at the timepoint where HFF infection with *N. caninum* tachyzoites was initiated, and incubated for 2 hours before removal by washing, there was a significant decrease in beta-galactosidase activity compared to experiments where compound 1266 [16] (negative, low-potency control) or DMSO were assessed (t-test; p<0.001) (Figure 3A). With subsequent addition of 1294 at 60, 90 and 115 minutes after initiation of HFF infection, there was no significant difference in beta-galactosidase activity, and no difference could be seen when 1294 was added after the infection phase of 2 hours had ended (p>0.2). SEM of HFF monolayers infected with *N. caninum* tachyzoites either in the presence or absence of compound 1294 (Figure 3B–D) and subsequently maintained in the presence of 1294 for 3 days showed that proliferation of parasites was markedly inhibited when the compound was added during the infection phase, but not when 1294 was added after infection has taken place. In other experiments involving *in vitro* 1294 treatment of infected HFF for up to 12 days, however, we could show that the effects mediated by 1294 are not only restricted to the inhibition of host cell entry. Continuous inspection by light microscopy showed that intracellular *N. caninum* tachyzoites did initially undergo proliferation in the presence of 2.5 μM 1294, but lysis of host cells was evident only in a few instances. However, from day 5 post infection onwards in the presence of 2.5 μM 1294, no extracellular tachyzoites were visible (data not shown). To assess whether any viable parasites remained, after 12 days of continuous 1294 treatment the infected HFF cultures were trypsinized and were further cultivated with a fresh monolayer in the absence of compound 1294. Some tachyzoites resumed proliferation and host cell lysis occurred again within 3 days, leading to complete lysis of monolayers within one week (data not shown). This indicated that the interference of compound 1294 with *N. caninum* development was not completely parasiticidal under the conditions used in this experiment. However, this experiment was not quantitative and did not distinguish whether a substantial fraction of the parasites were non-viable. To understand if the parasites were damaged by exposure to 1294 once intracellular, we initiated the following ultrastructural experiments.

### Effects of compound 1294 on *N. caninum* tachyzoite ultrastucture

**Figure 4:**
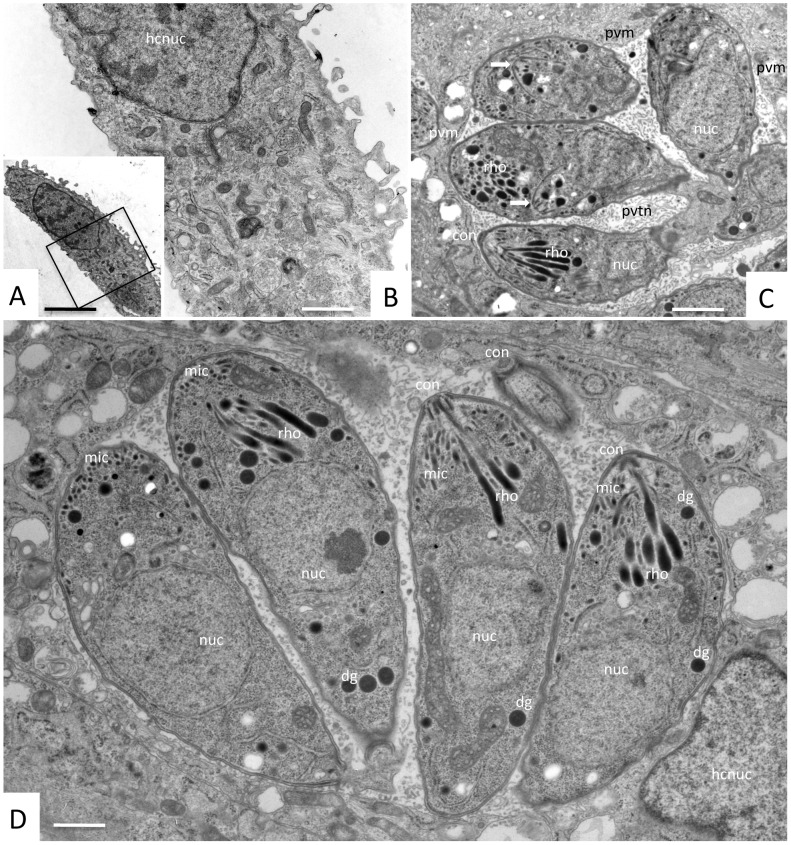
TEM analysis of compound 1294 treated and untreated *N. caninum*-infected HFF cultures at 3 days post-infection. **A** shows a HFF cell with inhibitor 1294 (2. 5 μM) added at the time point of infection, **B** represents a higher magnification view of A. Note the absence of parasites, and the fact that compound 1294 does not induce any obvious ultrastructural alterations within the host cell. **C** and **D** show *N. caninum*-infected HFF cultured in the absence of the inhibitor at 3 days post-invasion. Numerous tachyzoites are located within parasitophorous vacuoles, surrounded by a parasitophorous vacuole membrane (PVM), and embedded in a matrix of a tubular membrane network (PVTN). Apical parts of tachyzoites are characterized by the conoid (con), micronemes (mic), and rhoptries (rho); dg  =  dense granules, nuc  =  tachyzoite nucleus, hcnuc  =  host cell nucleus. The two arrows in C point towards proliferating tachyzoites undergoing endodyogeny. Bars in A = 5 μm; B = 1 μm; C = 0.58 μm; D = 0.28 μm.

TEM analysis of *N. caninum* infected HFF cultures confirmed that the effects of 1294 were not entirely restricted to invasion inhibition, but that at a concentration of 2.5 μM the compound also interfered in the intracellular development of *N. caninum* tachyzoites with no observable evidence of damage to the mammalian host cells. When the inhibitor was added at the time point of infection, basically no intracellular tachyzoites could be observed by TEM, and HFF morphology remained unaltered, confirming that compound 1294 did not affect the host cells but rather blocked tachyzoite invasion (Figure 3D, 4A and B). Transmission electron micrographs of untreated and *N. caninum*-infected host cells at 3 days after invasion are shown (Figure 4C and D). Tachyzoites are located within parasitophorous vacuoles (PVs), of varying sizes depending on the number of tachyzoites present, and they are separated from the cytoplasm by a parasitophorous vacuole membrane (PVM), and embedded in a tubular membrane network (PVTN).

**Figure 5:**
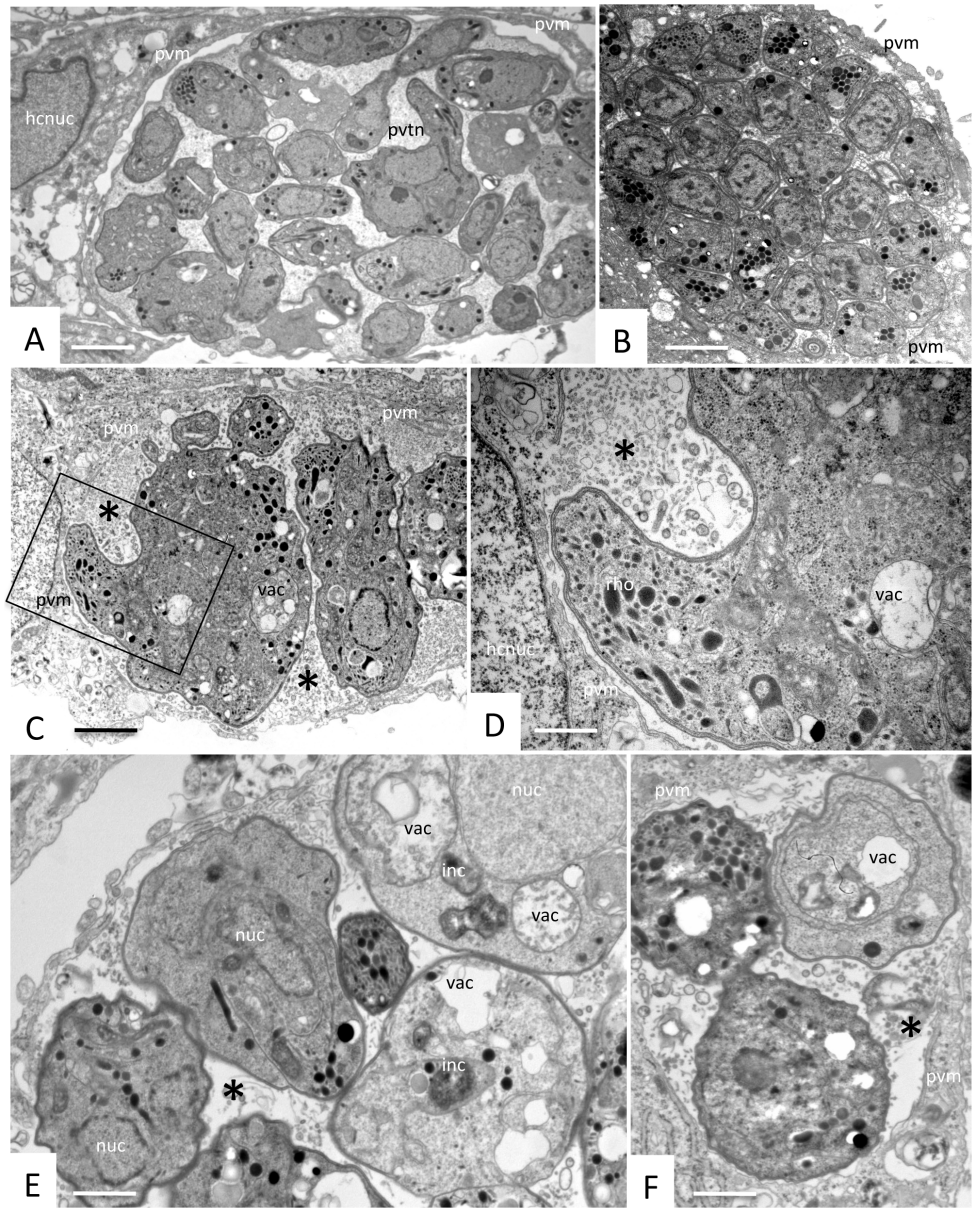
TEM analysis of *N. caninum*-infected HFF cultures treated for 3 days, with 2.5 μM of inhibitor 1294 added at 2 h post-infection. A and B show micrographs of more or less densely packed parasitophorous vacuoles containing numerous tachyzoites without obvious alterations. C and D show a representative example of a vacuole delineated by a parasitophorous vacuole membrane (pvm) containing parasites displaying a large cytoplasmic mass and aberrant overall morphology. The boxed area in C is enlarged in D, exhibiting the presence of the pvm and rhoptry-like organelles (rho). In many instances, as seen in Figure 5E and F, parasitophorous vacuoles contain several parasites exhibiting clear signs of metabolic impairment such as cytoplasmic vacuolization (vac) and electron-dense inclusions (inc). (Figure 5E, F). Note that in C–F the matrix has lost its characteristic tubular network structure and is now formed of either granular material or possibly membranous material (C, D), or is even largely missing (E. F). Bars in A = 1 μm; B = 0.9 μm; C = 0.75 μm, D = 0.35 μm; E = 0.3 μm; F = 0.3 μm.

**Figure 6:**
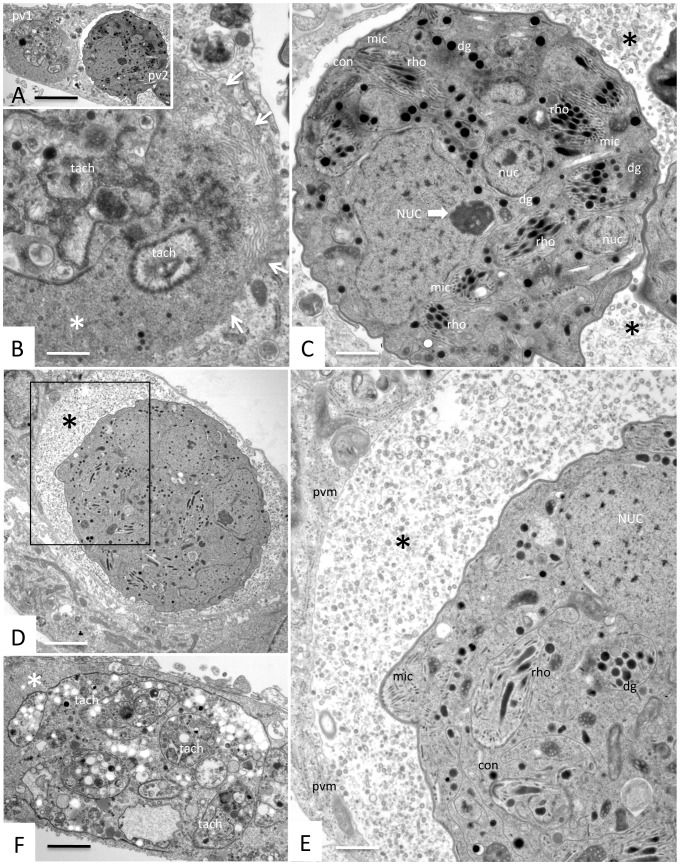
TEM analysis of *N. caninum-*infected HFF cultures in the presence of 2.5 μM of inhibitor 1294 for 5 and 9 days. **A–C** illustrate two distinct outcomes of treatment with 2.5(PV1 and PV2) in a HFF cell. In **B**, the higher magnification view of PV1 reveals non-viable remnants of tachyzoite (tach), now largely embedded in a solid matrix (*), and as indicated by arrows, the parasitophorous vacuole membrane has been replaced by a rather amorphous transition zone between PV1 and the host cell cytoplasm. In **C**, PV2 displays a complex of still viable and presumably proliferating, but non-separating, parasites forming a large multi-nucleated mass, with clearly discernable micronemes (mic), rhoptries (rho), dense granules (dg), and smaller nuclei (nuc). Note the large nuclear mass (NUC) in the center with a large nucleolus marked by the bold arrow. In **D**, and at a higher magnification in E, a similar multinucleated complex is shown after 9 days of treatment with compound 1294, also displaying a large nuclear mass (NUC), as well as rhoptries, micronemes, dense granules, and an intact parasitophorous vacuole membrane. In **F**, a non-viable complex with tachyzoite remnants (tach) is shown after 9 days of treatment. Note the difference in electron density of the matrix of PVs containing non-viable parasites (B, F) compared to viable multinucleated complexes (C, D, E). Bars in A = 5 μm; B = 0.8 μm; C = 0.9 μm; D = 2 μm; E = 0.7 μm; F = 0.9 μm.

When the compound was applied 2 hours after tachyzoites achieved host cell entry, a variety of effects can be visualized already after 3 days of treatment. On one hand, we observed parasitophorous vacuoles containing numerous and densely packed tachyzoites similar to the ones observed in untreated cultures (Figure 5A and B), indicating that the compound did not have any profound effects on the intracellular development of *N. caninum* tachyzoites. However, in many instances, vacuoles were seen that contained parasites with an enlarged cytoplasmic mass and aberrant overall morphology (Figure 5C and D), and also vacuoles containing typically a lower number of parasites exhibiting signs of metabolic impairment such as cytoplasmic vacuolization and electron-dense inclusions (Figure 5E and F). More pronounced alterations were visible at day 5 of drug treatment (Figure 6). On one hand, largely destroyed parasites were observed intracellularly, located still within a parasitophorous vacuole, but the matrix of this vacuole was becoming electron dense and granular in appearance, and the parasitophorous vacuole membrane was not clearly discernible anymore (Figure 6A and B). On the other hand, numerous infected HFF contained complexes of still viable and presumably proliferating, but non-separating, parasites forming a large multi-nucleated mass within a parasitophorous vacuole clearly delineated and separated from the host cell cytoplasm by a membrane. Interestingly these complexes often contained several smaller nuclei in addition to an outstandingly enlarged nucleus with a respectively enlarged nucleolus (Figure 6C). The two contrasting phenomena were sometimes seen within the same host cell (Figure 6A). At day 9 of 1294 treatment, these multinucleated complexes increased in size, and the matrix of the parasitophorous vacuole has completely lost the characteristics of a tubular membranous network and was replaced by components of more granular and vesicular appearance (Figure 6D and E). In many instances, however, the ultrastructure of these parasite complexes was severely damaged and only residual, non-viable complexes were seen, located within an electron-dense matrix. Under the conditions used here, the cultures were completely lysed at time points later than day 4, so it was impossible to show a control, because this will contain mostly extracellular (and dying) parasites. Taken together, these results clearly indicate that compound 1294 does not only affect host cell invasion, but that this compound does also interfere in separation of zoites once nuclear division and cytoplasmic duplication has taken place, resulting in the appearance of large, multi-nucleated masses, which are, however, still viable. Once the compound is removed, these effects are most likely reversible, and division of tachyzoites proceeds, resulting in lysis of the host cells.

### Effects of 1294 on *N. caninum* infected Balb/c mice

**Figure 7:**
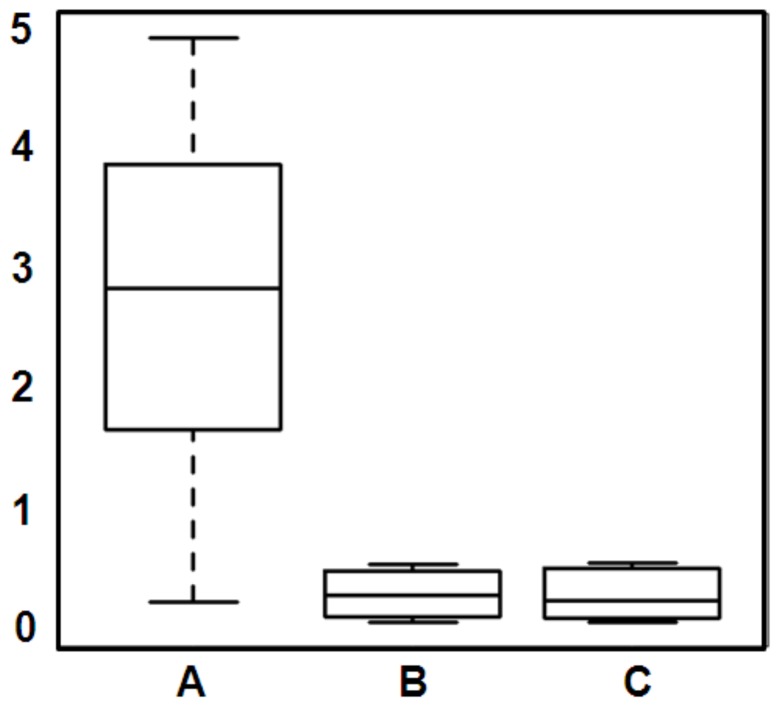
Compound 1294 treatment reduces cerebral parasite load in *N. caninum* infected mice. Balb/c mice (8 animals per experimental group), infected intraperitoneally with 5×10^6^ *N. caninum*-β-gal tachyzoites, were treated with compound 1294 (50 mg/kg in 100 μL honey suspension). Group A (placebo) received honey for 5 days, group B received 1294 treatment starting at 3 days post infection and continuing daily for 5 days; group C received the same as group B but for 10 days. None of the mice exhibited clinical signs at 28 days post-infection, after which the cerebral parasite load was assessed by quantitative real time PCR.

In the murine experimental model of infection, as expected, none of the mice showed abnormal behavior or clinical signs of neosporosis during the entire 28 days of experiment. Treatment with 1294 led to a reduction of the cerebral parasite load in mice previously infected by *N. caninum* as determined by quantitative RT-PCR (shown in Figure 7). Cerebral parasite burdens in the different groups were analyzed by ANOVA after logarithmic transformation of the original data. ANOVA revealed that values from at least one group were statistically different from another (p<0.01) followed by post-hoc test according to Bonferroni to compare the groups B and C to the control group A. Amongst the 2 experimental treatment regimens, the one applied to group C (1294 therapy starting on day 3 post infection and continued for 10 days) was clearly the most effective (*P* = 0.0047). Treatment group B (treatment as in C but only for 5 days) also showed a tendency towards reduced parasite load (*P* = 0.0139). Treatment of 1294 did not inhibit infection of the brain completely. Nevertheless, the degree of efficacy in this model of infection was particularly significant when considering that treatment with 1294 was discontinued >2 weeks before the end of the experiment and collection of the brains for qPCR and yet minimal numbers of parasites were observed in the treated groups (Figure 7).

## Discussion

*Toxoplasma gondii* and *Neospora caninum* are obligate intracellular parasites that must invade the host cell to grow and replicate. Proliferation occurs after successful invasion of a host cell by a complex process of coordinated secretion of proteins regulated in part by changes in intracellular calcium concentrations. Cell invasion, in this case, is independent of host calcium control systems but relies on a calcium-regulated secretion pathway in the parasite [28], [29] directed in part by CDPKs. This points to an autonomous system with heavy reliance on the parasites' intrinsic factors, an intriguing opportunity for anti-apicomplexan specific chemotherapy. *Toxoplasma gondii* calcium-dependent protein kinase 1 (*Tg*CDPK1) has generated increasing interest in recent years because of its essential physiological roles and as a potential drug development target [15], [22], [30]–[32]. A high degree of amino acid sequence and structural similarities between *Toxoplasma gondii* and *Neospora caninum* CDPK1 enzymes and the high degree of conservation between *T. gondii* and *N. caninum* genomes suggests that both enzymes have similar functional characteristics. By extrapolating from *Tg*CDPK1 functional characteristics, it would be anticipated that inhibitors of *Nc*CDPK1 would be sufficient to interrupt the growth and proliferation of *N. caninum* tachyzoites by preventing host cell invasion and/or egress. Nonetheless, potential functional divergence between *Tg*CDPK1 and *Nc*CDPK1 combined with the distinct range of mammalian host systems in which the parasites operate warrants detailed understanding of possible differences in their molecular structure and specific phenotypic response to their inhibition by BKIs.

This study presents the first crystal structures of parasitic protozoan *N. caninum* CDPK1 enzyme. The inhibitor structure-activity relationships (SARs) of *Nc*CDPK1 and *Tg*CDPK1 for all compounds tested are virtually identical (Figure 1) [22] suggesting that similar BKIs could be useful for both toxoplasmosis and neosporosis therapy. This observation supports our inhibitor-binding mode model derived from BKIs-*Tg/Nc*CDPK1-complex crystal structures that shows favorable interaction of the C3 aryl bump with the gatekeeper hydrophobic pocket (Figure 2A, Figure S1 and S2 in File S1). Earlier observations strongly suggest that the vicinity of the ribose pocket can serve as an additional potency and selectivity-determining region especially in BKI analogues with a piperidine R_2_ group [22], [24]. However, *Nc*CDPK1 has a similar but not identical pose as the piperidine R2 substituent in the ribose pocket relative to *Tg*CDPK1 (Figure 2A, Figure S1 and S2 in File S1). Furthermore the small-angle X-ray scattering (SAXS) analysis presented in this study constitutes the first direct characterization of the calcium-responsive conformational rearrangement of CDPK1 in solution. As previously found for the structurally similar calcium-binding domain of calmodulin, the calcium-bound form of CDPK1 observed in solution is less compact than the calcium-bound form of the same protein observed crystallographically. The implied multiplicity of calcium-bound states is entirely compatible with our understanding of the mechanism of calcium-regulated kinase activity, in which the calcium-induced rearrangement displaces structural elements that limit access to the active site. However it indicates that previous crystal structures of CDPK1 calcium-bound states do not represent a single biologically active conformation present in solution. By contrast, the SAXS profile for the calcium-free state in solution is consistent with the crystal structures of the calcium-free state of *Tg*CDPK1 and *Nc*CDPK1 used to interpret structure-activity relationships. Thus structural studies of CDPK enzymes in complex with BKIs together with computational modeling have been useful in guiding synthetic chemistry for lead optimization by predicting functional groups needed to improve potency, selectivity and further optimize PK/ADME properties.

We have experimental evidence that *N. caninum* is prevented from invading the mammalian host cell when lead compound 1294 is added at the time of infection. By EM and light microscopy, some parasites treated with 1294 after infection appear viable, but appear to be unable to lyse the HFF host cells. Absence of any extracellular tachyzoites 5 days after 1294 treatment of infected HFF culture is consistent with inhibition of *N. caninum* egress. In normal cell culture, *N. caninum* tachyzoites lyse or egress from their host cells at around 72 hours (3 days) after infection [19], [33]. BKI inhibition of *Tg*CDPK1 have been linked to the process of invasion and egress of the mammalian host [31], [34], thus what we are observing in *N. caninum* is consistent with that observation in *T. gondii*.

Beside potential impediment of egress, TEM analysis of HFF cultures infected with *N. caninum* tachyzoites prior to treatment with compound 1294 for an extended length of time (up to 12 days), revealed major ultra-structural alterations in *N. caninum* tachyzoites, but not in mammalian host cells. More precisely, the data suggests additional effects of BKIs on molecular target(s) that control(s) separation of tachyzoites during replication, which results in either parasites dying off intracellularly, or seemingly viable parasites forming large multinucleated complexes. In other instances, these complexes also show signs of metabolic impairment. To the best of our knowledge, there is no literature or experimental evidence of *Nc*CDPK1 or *Tg*CDPK1 involvement in transducing signals needed in metabolic pathways or cell division. However, egress impediment induced stress from 1294 treatment of *N. caninum* tachyzoite infected HFF cells could be a potential indirect cause or unrelated to inhibition of *Nc*CDPK1. It is also possible that the difference in potency or phenotypic effect on *N. caninum* versus *T. gondii* is due to the presence of a second target kinase in *N. caninum*. Comparison of the respective genomes argues against this, however. The most plausible off-target activity would be recognition of another small-gatekeeper kinase. Seven such kinases can be found in the *N. caninum* genome, but in each case the protein is directly homologous (E value  = e∧-100 or better) to a *T. gondii* kinase whose sequence contains that same small gatekeeper residue. While this does not rule out the possibility of off-target activity, on balance, it seems more likely that *N. caninum* is somewhat more sensitive than *T. gondii* to interference with the targeted CDPK1. Previous study on ruthenium complexes had shown that drug effects can clearly differ between *T. gondii* and *N. caninum* [35]. Similarly, these observations may be further phenotypic characteristics of *Nc*CDPK1 inhibition suggesting a potential functional divergence or an amplification of physicochemical role relative to *Tg*CDPK1. When infected HFF were treated with 1294 for 12 days, followed by passage by trypsinization to remove the compound, proliferation of tachyzoites resumed, indicating that the drug is not parasiticidal *in vitro*. Rather the trypsin may release the viable tachyzoites, which can re-establish infection. However, it is unclear what specific percentage of intracellular tachyzoites was damaged due to the prolonged exposure to 1294; it is only clear that some of the tachyzoites are viable and can re-establish infection.

Compound 1294 was recently shown to be a highly selective inhibitor for parasites' CDPKs compared with 80 mammalian kinases [23] presumably due to smaller gatekeeper residues in CDPKs and other interactions in the CDPKs active sites [24]. Other enzymes in the *N. caninum* kinome with small gatekeeper residues, that compound 1294 could potentially inhibit, include *Nc*CamK (NCLIV_046430) with an alanine gatekeeper, *Nc*AGC like kinase (NCLIV_016060) with a serine gatekeeper, *Nc*ROP like kinase (NCLIV_030990) with a threonine gatekeeper, and *Nc*MAPK-3 (NCLIV_056080) with a serine gatekeeper. *Nc*MAPK-3 is a *Tg*MAPK-1 (TGME49_312570) ortholog with highly conserved kinase domain of >80% identity. *Tg*MAPK-1 been shown to play some role in sister parasite *T. gondii* stress response [36], cell division [37]. It is unclear to what extent compound 1294 could impact these targets, but sensitivity is postulated to be related to the size of the respective enzymes' gatekeeper residue in the order alanine>serine>threonine. Intracellular damage to tachyzoites may be concentration dependent probably due to different levels of sensitivity of prospective *N. caninum* off-target enzymes to compound 1294.

In addition to demonstrating the molecular and structural basis of BKIs impact on the growth of *N. caninum* tachyzoites in cell culture, this study provides essential fundamental evidence for potential clinical relevance of 1294 in the management of *N. caninum* infection. Although the *in vitro* data suggest reversible effects on growth inhibition with the removal of drug pressure, the clinical outcome in a mouse model of infection indicates that five to ten days treatment results in an almost undetectable cerebral parasite burden with appropriate length of treatment (Figure 7). Barely detectable number of parasites in the brains of mice from 1294-treated groups at least 2 weeks after the end of treatment, compared to the high levels of brain parasites in the control group, strengthens the argument that 1294 is efficacious *in vivo* despite not being parasiticidal in *in vitro*. This is consistent with recent observation of 1294 efficacy in the mouse cryptosporidiosis model [17]. Although we cannot prove there was no regrowth of *N. caninum* in the mouse model after 1294 dosing, the very low parasite counts over 2 weeks after dosing with 1294 (Figure 7) is good evidence that *in vivo* regrowth was minimal or immunologic containment was established after the end of dosing. The reduced numbers of *N. caninum* tachyzoites in the brains of treated mice is evidence that compound 1294 crosses the blood-brain barrier in sufficient concentration to be useful in clinical cases. Indeed, our unpublished studies have shown that 25 to 33% of the plasma levels of 1294 can be found in brain tissue of mice given a single dose of 1294. Favorable pharmacokinetic and pharmacodynamic properties of compound 1294 [23] combined with an immunologic response, is likely to facilitate a more effective and complete clearance of parasites *in vivo* compared to the *in vitro* situation.

Overall, the prospect of a broad-spectrum anti-apicomplexan agent with no mammalian toxicity profile is quite exciting. Although further studies will be needed to fully elucidate the molecular target(s) of 1294, it seems that its role of inhibiting the host cell invasion and egress process is only one of its many functions.

## Materials and Methods

### Cloning and Protein Production

A truncated coding region of *cdpk1* (NCLIV_011980) gene (nucleotide base 64−1521) was amplified from *N. caninum* Liverpool strain cDNA using the primers *LICNcCDPK1_G23Fwd* (5′- GGG TCC TGG TTC GGG GGC CGC CGG TGG AGC GGG AGA CAA GCT CCA TGC GAC G -3′) and *LICNcCDPK1_Rv* (5′- CTT GTT CGT GCT GTT TAT TAG TTT CCG CAA AGC TTC AGG AGC ATT TGC TGA AAC -3′). The PCR product was cloned into the ligation independent cloning (LIC) site of expression vector pAVA0421 containing a cleavable 3C protease N-terminal 6-histidine tag and expressed in Rosetta *Escherichia coli* (DE3) strain (Novagen, USA) using Studier auto-induction protocols at 20°C [38]. Soluble recombinant protein was purified on a Ni^2+^-NTA affinity (Qiagen, Valencia, CA) column in binding buffer composed of 20 mM HEPES pH 7.25, 500 mM NaCl, 5% glycerol, 30 mM imidazole, 0.5% CHAPS, and 1 mM TCEP. The protein was eluted with the same buffer supplemented with 250 mM imidazole and further purified by size-exclusion chromatography as previously described [39].

### Activity Assay

Protein kinase activity of recombinant *Nc*CDPK1 and inhibition of its kinase phosphorylation properties by BKIs was measured in a non-radioactive assay using Kinase-Glo luciferase reagent (Promega, Madison, WI). This luminescence based assay measures kinase activity in the presence or absence of inhibitors by reporting changes in initial ATP concentration after *Nc*CDPK1 phosphorylation of biotinylated Syntide-2 peptide substrate (Biotin-C6-PLARTLSVAGLPGKK) (BioSyntide-2) (American Peptide Company, Inc. Sunnyvale, CA) [15], [40]. Enzyme activity assays in the presence of 40 μM BioSyntide-2 and 3.3 nM recombinant NcCDPK1 with or without inhibitor(s) were performed in a buffered solution containing 20 mM HEPES (pH 7.5), 0.1% BSA, 10 mM MgCl2, 1 mM EGTA, 2 mM CaCl2. The reaction was initiated by the addition of 10 μM ATP and incubated for 90 minutes at 30°C. Changes in initial ATP concentration was evaluated as a luminescence readout using a MicroBeta2 multi-label plate reader (Perkin Elmer, Waltham, MA). Results were converted to percent inhibition and IC_50_ values (the concentration of compound that led to 50% inhibition of enzyme activity) were calculated using non-linear regression analysis in GraphPad Prism (GraphPad Software, La Jolla, CA) [16], [22].

### Protein Crystallization

Diffraction quality crystals of *Nc*CDPK1 were grown after protein was gathered by Ni^2+^-NTA affinity chromatography, treated with 20 mM EGTA (incubated on ice for 1 hour), and further purified by size exclusion and an anion exchange column. The protein solution used for crystallization contained 25 mM HEPES pH 7.0, 500 mM NaCl, 5% glycerol, 5 mM DTT, 0.025% azide (protein buffer), 20 mM EGTA and ∼3 mg ml^−1^ protein. Crystals were grown by vapor-diffusion equilibration against a reservoir containing 0.2 M ammonium sulfate, 0.1 M Bis Tris (pH 5.1–5.6), 25–31% (v/v) PEG 3350 and 5 mM DTT. For growing inhibitor co-crystals, 20 mM inhibitor in 100% DMSO was diluted in 0.5 M EGTA and protein buffer, and subsequently mixed with protein solution to a final concentration of 200 μM inhibitor, 20 mM EGTA and 1% DMSO. One microliter (1 μL) protein-inhibitor solution and 1 μL reservoir solution were mixed for crystal growth at room temperature. In order to find optimal growth conditions a matrix was used with steps of 1% difference in PEG 3350 concentration and of 0.1 pH unit.

### X-ray Diffraction and Crystal Structure Determination

Crystals of *Nc*CDPK1 alone and cocrystallized in complex with inhibitors formed in space group P2_1_ with nearly isomorphous lattice packing. Diffraction images were collected at SSRL beamline 12-2 using X-rays with wavelength 0.9794 Å and processed using the programs Mosflm [41] and Aimless [42]. The apo *Nc*CDPK1 crystal diffracted to a resolution of 2.05 Å. Crystals of inhibitor complexes diffracted to resolutions between 2.6 Å and 3.1 Å. An initial structural model was developed by molecular replacement starting from a structure of the homologous CDPK1 from *T. gondii* followed by alternating manual rebuilding in Coot [43] with automated refinement in Refmac [44]. The refined model of the apo structure had crystallographic residuals R = 0.20 R_free_ = 0.26, with no residues in disallowed regions of conformational space (φ;ψ). Individual structural models for the lower resolution inhibitor complexes were developed by manually adjusting secondary structure elements to account for imperfect isomorphism followed by automated refinement in Refmac using strong “jellybody” restraints in order to maintain the good stereochemistry of the higher resolution starting model. The inhibitors were placed into difference electron density at the active site and included with the protein model during the final round of refinement. Crystallographic statistics for data and model quality for the structures of apo *Nc*CDPK1and complexes with inhibitors RM-1–132 (15n) [22] and 1294 (15o) [22] are given in Table S1 and Figure S1 and S2 in File S1. The corresponding PDB accession codes for these structures are 4m97, 4mxa, and 4mx9.

### Small Angle X-ray Scattering

Solution small angle X-ray scattering (SAXS) was measured from *Nc*CDPK1 at concentrations of 0, 3, 6, and 12 mg/ml in a buffer containing 500 mM NaCl, 5% glycerol, 25 mM HEPES and either 1 mM CaCl_2_ or 1 mM EGTA. The scattering experiments were performed at SSRL beamline 4–2 using 11 keV X-rays and a sample-to-detector distance of 170 cm. No evidence for protein aggregation in either the low or high calcium condition was found from comparing curves at different protein concentrations. The SASTBX package [45] was used to analyze scattering curves and to derive a low-resolution model for the conformations of the calcium-bound and calcium-free states in solution. We have initiated a deposition request for the SAXS data to the Bioisis project.

### *In vitro* efficacy studies

#### Short term assays - simultaneous infection and BKI-treatments

Transgenic *N. caninum* tachyzoites expressing beta-galactosidase under a constitutive promoter (*N. caninum*-β-gal) were grown in Vero cells and were separated from host cell components as previously described [46]. Human foreskin fibroblasts (HFF) at 5×10^3^ cells per well in phenol-red free culture medium were incubated at 37°C with 5% CO_2_ and were grown to confluence in a 96-well plate. Just prior to infection, the inhibitors were added at a final concentration of 2.5 μM for initial experiments and ranging between 0.5 nM and 2.5 μM for EC_50_ determinations (the effective concentration to reduce proliferation by 50%), or DMSO as a control. Cultures were infected with *N. caninum*-β-gal tachyzoites (1×10^3^ per well). After three days at 37°C/5% CO_2_, plates were centrifuged at 500 g, medium was removed, and cell cultures were lysed in PBS containing 0.05% Triton-X-100. After addition of 10 μL of 5 mM chlorophenol red-b-D-galactopyranoside (CPRG; Roche Diagnostics, Rotkreuz, Switzerland) dissolved in PBS, the absorption shift was measured at 570 nm wavelength at various time points on a VersaMax multiplate reader (Bucher Biotec, Basel, Switzerland). The activity, measured as the release of chlorophenol red over time, was proportional to the number of life parasites down to 50 per well as determined in pilot assays. EC_50_ values were calculated after the logit-log-transformation of relative growth and subsequent regression analysis by the corresponding software tool contained in the Excel software package (Microsoft, Seattle, WA) [46].

#### Short term assays – BKI treatments pre- and post-infection

A subsequent experiment to determine the effects of lead BKI compound 1294 on *N. caninum* invasion of mammalian cells was performed. Here, HFF cell monolayers grown in 96 well plates were infected with 2×10^4^ tachyzoites and treated with compounds 1294 or 1266 [16] (as a BKI control that inhibited *Nc*CDPK1 >250-fold less efficiently than 1294) at a final concentration of 5 μM per well at the time of initiation of infection, as well as after 60, 90 and 115 minutes after addition of tachyzoites. Control wells with equal volume of DMSO were also included per reaction plate. Two hours after initiating the experiment, the cells were washed. Selected untreated assay plates were treated with 1294 for 60 minutes after the initial wash with medium. All the assay plates were incubated for one hour before a final wash and reading the beta-galactosidase activity.

#### Long term treatment with compound 1294

HFF monolayers were grown in 24 well tissue culture flasks, and were infected with 1×10^4^ *N. caninum* tachyzoites for 2 hours, followed by addition of compound 1294 (2.5 μM). Prolonged treatments for up to 12 days were carried out, with medium changes and addition of fresh drug every 3 days. For some experiments, 1294-treated *N. caninum*-infected monolayers were trypsinized after 12 days and cultures were split into two, one further maintained in the presence of 1294 and the other one in the absence of the compound for up to 5 days. For transmission electron microscopy (TEM), similar assays were carried out using HFF monolayers grown in T25 tissue culture flasks and infected with 5×10^5^ *N. caninum* tachyzoites. After selected time points, cells were fixed and processed as described below.

### TEM analysis of *N. caninum*-infected HFF treated with compound 1294

Infected and 1294-treated cultures maintained in T25 tissue culture flasks were processed for TEM at different time points after infection (1, 3, 5 and 9 days) as described earlier [47], [48]. Infected cultures with no compound treatment were used as controls. Fixation was carried out in 100 mM sodium cacodylate buffer (pH 7.3) for 2 hours at room temperature, followed by post-fixation in 2% osmium tetroxide in cacodylate buffer for 2 hours. Following washes in water and pre-staining in saturated uranyl acetate solution in water for 30 minutes, specimens were rinsed in water, dehydrated in ethanol and embedded in Epon 812 resin as previously described [19], [20]. The resin was polymerized at 60°C overnight, and ultrathin sections were cut on a Reicher & Jung microtome, placed onto a formvar-coated grid and stained with uranyl acetate and lead citrate. Specimens were viewed on a Phillips EM 400 transmission electron microscope operating at 60 kV.

### In vivo effects of 1294 treatment in *N. caninum* infected Balb/c mice

Twenty-four female Balb/c mice between 8 and 9 weeks of age were purchased from Charles River Laboratories (Sulzheim, Germany) and were maintained in a common room under controlled temperature and a 14 hour dark/10 hour light cycle with food and water *ad libitum* according to the animal welfare legislation of the Swiss Veterinary Office. At day zero, mice were randomly caged into 3 experimental groups of 8 mice. Enzyme-linked immunosorbent assay (ELISA) was carried out to ensure that mice were serologically *Neospora*-negative [49]. Compound 1294 was initially formulated in honey for oral application as described in Küster *et al* [50], at a dose of 50 mg/kg in 100 μL suspension for both prophylactic and post-infection treatments. The following oral treatments by standard gavage were carried out: Experimental group A received honey only (daily placebo treatment for 5 days), starting at 2 hours prior to infection; group B received 1294 treatment starting at 3 days post infection and continuing daily for 5 days; group C received the same as group B but for a duration of 10 days. Infection was carried out by intra-peritoneal inoculation of 5×10^6^ freshly isolated *N. caninum*-β-gal tachyzoites in 100 μL medium. The experiments were terminated 28 days after infection. Mice were euthanized, brain tissues were collected, and the cerebral parasite load was evaluated by quantitative real time-PCR specific for *N*. *caninum* [46], [49].

### Statistics

Statistical analysis of the results was performed with suitable tools from the open source software package R. Differences exhibiting p values of <0.01 were considered significant. IC_50_ values were calculated after the logit-log-transformation of the relative growth (RG; control = 1) according to the formula ln (RG/(1-RG)) = a×ln(drug concentration) +b followed by regression analysis.

### Ethics Statement

Human foreskin fibroblasts (HFF; ATCC SRC-1041) were purchased from the American Type Culture Collection (Manassas, VA, USA, http://www.atcc.org/products/all/SCRC-1041.aspx). Experimental animals were maintained according to the animal welfare legislation of the Swiss Veterinary Office and Institutional Animal Care and Use Committees (IACUC). Facilities and procedures for maintenance of experimental animals are fully accredited by the American Association for Laboratory Animal Care (AALAC) and approved by the University of Washington IACUC under protocol number 2154-01.

## Supporting Information

File S1 **Combined file of supporting figures and tables.** Table S1: Data collection and refinement statistics. Figure S1: Electron density for compound 1294 (15o) in the active site of *Nc*CDPK1. Green density cages are difference electron density from an initial map calculated prior to adding the inhibitor to the structural model (3 sigma contours). Blue density cages are mFo-Fc density after refinement of the protein and inhibitor. Figure S2: Electron density for compound RM-1-132 (15n) in the active site of *Nc*CDPK1. Green density cages are difference electron density from an initial map calculated prior to adding the inhibitor to the structural model (3 sigma contours). Blue density cages are mFo-Fc density contoured at 2.5 sigma after refinement of the protein and inhibitor. The faint purple trace is a superposition of the same compound as bound in the active site of the homologous *Tg*CDPK1.
(DOCX)
