# Supplementary material for: Neospora caninum Calcium-Dependent Protein Kinase 1 Is an Effective Drug Target for Neosporosis Therapy
Source: PLoS One. 2014 Mar 28;9(3):e92929. doi: 10.1371/journal.pone.0092929 (PMC3969379; doi:10.1371/journal.pone.0092929)

**Supplementary information**

***Neospora caninum* calcium-dependent protein kinase 1 is an effective drug target for neosporosis therapy**

Kayode K. Ojo^1#^, Molly C. Reid^1^, Latha Kallur Siddaramaiah^2^, Joachim Müller^3^, Pablo Winzer^3^, Zhongsheng Zhang^2^, Katelyn R. Keyloun^1^, Rama Subba Rao Vidadala^4^, Ethan A. Merritt^2^, Wim G.J. Hol^2^, Dustin J. Maly^4^, Erkang Fan^2^, Wesley C. Van Voorhis^1#^, Andrew Hemphill^3#^

^1^Center for Emerging and Re-emerging Infectious Diseases (CERID), Division of Allergy and Infectious Diseases, Department of Medicine, University of Washington, Seattle, Washington, United States of America

^2^Department of Biochemistry, University of Washington, Seattle, Washington, United States of America

^3^Institute of Parasitology, Vetsuisse Faculty, University of Berne, Berne, Switzerland.

^4^Department of Chemistry, University of Washington, Seattle, Washington, United States of America

# Address correspondence to

Kayode K. Ojo

[ojo67kk@u.washington.edu](mailto:ojo67kk@u.washington.edu)

Andrew Hemphill

andrew.hemphill@vetsuisse.unibe.ch

Wesley C. Van Voorhis

wesley@uw.edu

**Keywords:** Apicomplexa, *Neospora* *caninum*, calcium-dependent protein kinase, drug target, bumped kinase inhibitors.

**Supplementary information**

**Table S1**: **Data collection and refinement statistics**

|  |  |  |  |
| --- | --- | --- | --- |
|  | apo | RM-1-132 | 1294 |
| **PDB entry** | **4m97** | **4mxa** | **4mx9** |
| **Space Group** |  |  |  |
| **Unit Cell (a b c Å)** | 48.06 72.69 65.75 | 48.22 72.53 65.65 | 48.38 73.14 66.27 |
| **(**αβγ **°)** | 90 96.98 90 | 90 99.28 90 | 90 99.84 90 |
| **Wavelength (Å)** | 0.9794 | 0.9794 | 0.9794 |
| **Resolution (Å)** | 65-2.05 (2.11-2.05) | 48-3.0 (3.21-3.00) | 65-3.10 (3.31-3.10) |
| **Total number of unique reflections** | 27308 (2149) | 8027 (1432) | 7664 (1404) |
| **Replicate correlation** | 0.74 (0.40) | 0.68 (0.19) | 0.93 (0.29) |
| **Completeness (%)** | 97 (98) | 89 (90) | 92 (93) |
| **I/**σ**(I)** | 6.3 (0.9) | 3.6 (1.0) | 4.5 (1.5) |
| **Redundancy** | 3.9 (3.9) | 2.8 (2.7) | 2.6 (2.6) |
| **Contents of asymmetric unit** | 1 monomer | 1 monomer | 1 monomer |
|  |  |  |  |
| **Refinement Resolution (Å)** | 60-2.05 | 48-3.00 | 49-3.10 |
| **R** | 0.203 | 0.252 | 0.249 |
|  | 0.263 | 0.278 | 0.284 |
| **RMSD bonds (Å)** | 0.011 | 0.011 | 0.012 |
| **RMSD angles (°)** | 1.439 | 1.430 | 1.463 |
| **Protein atoms** | 3756 | 3711 | 3711 |
| **Non-protein atoms** | 128 | 56 | 55 |
| **Residues in favored regions (%)** | 97 | 96 | 96 |
| **Residues in disallowed regions** | 0 | 2 | 2 |
| **TLS groups** | 6 | 6 | 6 |
| **Mean protein atoms ()** | 25.9 | 49.2 | 50.3 |
| **Mean non-protein atoms ()** | 47.9 | 34.3 | 42.8 |

**Figure S1:** Electron density for compound 1294 (15o) in the active site of *Nc*CDPK1.


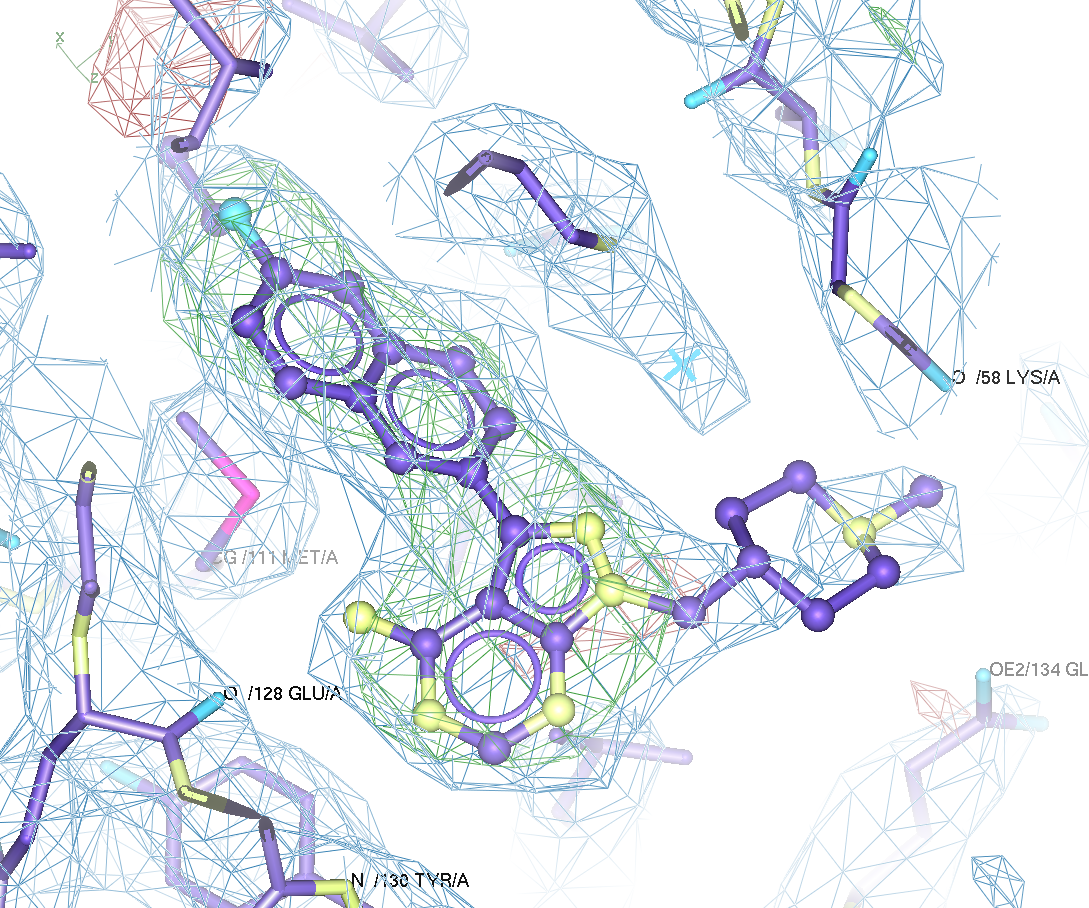


**Figure S2:** Electron density for compound RM-1-132 (15n) in the active site of *Nc*CDPK1


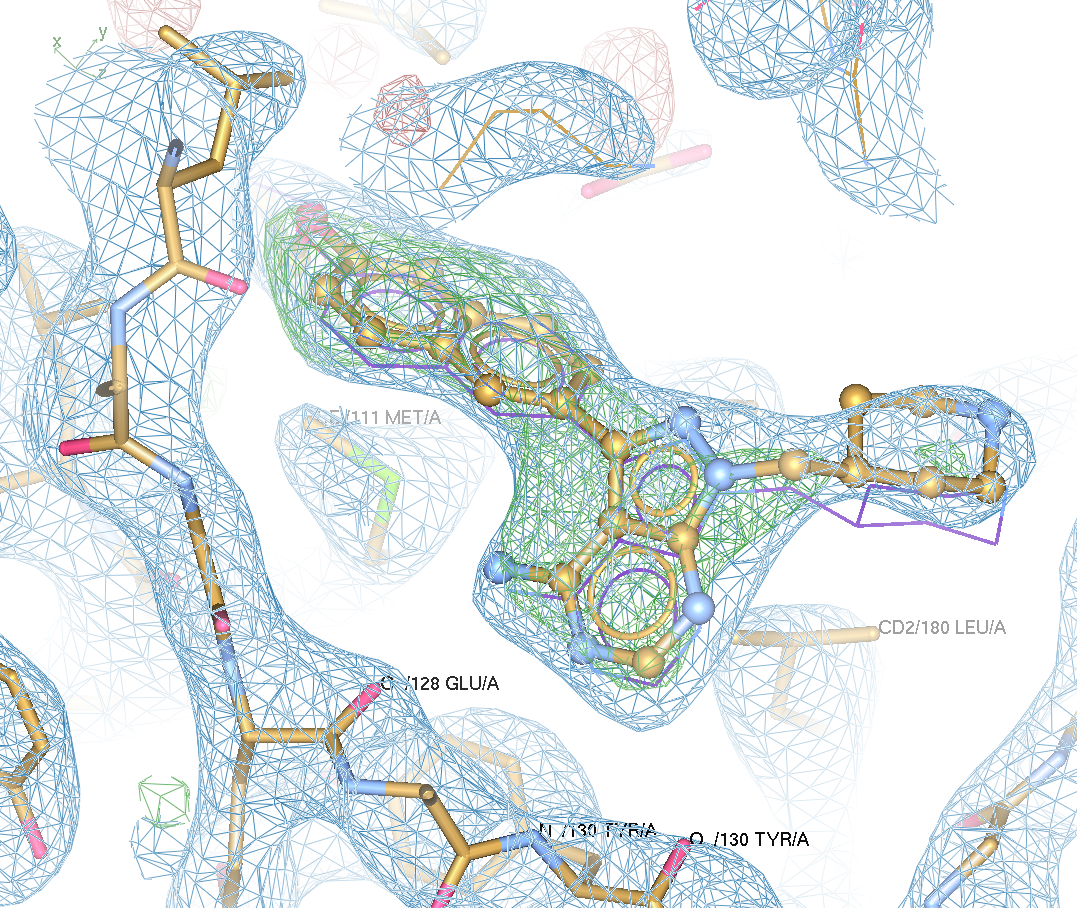

Supplement: File S1 — Combined file of supporting figures and tables. Table S1: Data collection and refinement statistics. Figure S1: Electron density for compound 1294 (15o) in the active site of NcCDPK1. Green density cages are difference electron density from an initial map calculated prior to adding the inhibitor to the structural model (3 sigma contours). Blue density cages are mFo-Fc density after refinement of the protein and inhibitor. Figure S2: Electron density for compound RM-1-132 (15n) in the active site of NcCDPK1. Green density cages are difference electron density from an initial map calculated prior to adding the inhibitor to the structural model (3 sigma contours). Blue density cages are mFo-Fc density contoured at 2.5 sigma after refinement of the protein and inhibitor. The faint purple trace is a superposition of the same compound as bound in the active site of the homologous TgCDPK1. (DOCX) [file pone.0092929.s001.docx]
